# Embedding data provenance into the Learning Health System to facilitate reproducible research

**DOI:** 10.1002/lrh2.10019

**Published:** 2016-12-27

**Authors:** Vasa Curcin

**Affiliations:** ^1^ Division of Health and Social Care Research King's College London London UK; ^2^ Department of Informatics King's College London London UK

**Keywords:** data provenance, health informatics, reproducibility

## Abstract

**Introduction:**

The learning health system (LHS) community has taken up the challenge of bringing the complex relationship between clinical research and practice into this brave new world. At the heart of the LHS vision is the notion of routine capture, transformation, and dissemination of data and knowledge, with various use cases, such as clinical studies, quality improvement initiatives, and decision support, constructed on top of specific routes that the data is taking through the system. In order to stop this increased data volume and analytical complexity from obfuscating the research process, it is essential to establish trust in the system through implementing reproducibility and auditability throughout the workflow.

**Methods:**

Data provenance technologies can automatically capture the trace of the research task and resulting data, thereby facilitating reproducible research. While some computational domains, such as bioinformatics, have embraced the technology through provenance‐enabled execution middlewares, disciplines based on distributed, heterogeneous software, such as medical research, are only starting on the road to adoption, motivated by the institutional pressures to improve transparency and reproducibility.

**Results:**

Guided by the experiences of the TRANSFoRm project, we present the opportunities that data provenance offers to the LHS community. We illustrate how provenance can facilitate documenting 21 CFR Part 11 compliance for Food and Drug Administration submissions and provide auditability for decisions made by the decision support tools and discuss the transformational effect of routine provenance capture on data privacy, study reporting, and publishing medical research.

**Conclusions:**

If the scaling up of the LHS is to succeed, we have to embed mechanisms to verify trust in the system inside our research instruments. In the research world increasingly reliant on electronic tools, provenance gives us a lingua franca to achieve traceability, which we have shown to be essential to building these mechanisms. To realize the vision of making computable provenance a feasible approach to implementing reproducibility in the LHS, we have to provide viable mechanisms for adoption. These include defining meaningful provenance models for problem domains and also introducing provenance support to existing tools in a minimally invasive manner.

## 1 INTRODUCTION

Our world is increasingly driven by data. Medical, economic, and political decisions are made based on automated analysis of ever‐growing volumes of data, be they patient treatment decisions generated from rule models or stock trading decisions made by microtrading tools. Scientific discovery is now all but impossible without data‐intensive infrastructures,^1^ which have transformed both how science is done and what science has done.^2^ But, in this fresh landscape, there needs to be an increased focus on the quality of data and research tasks, since new technological advances and cultural paradigms may bring down the control zones that existed to ensure the quality of scientific data and the research process.^3^ More generally, growth in size and complexity of data and analytics surrounding it form a black box around the reasoning behind important results and decisions. Understanding the provenance of the data and processes that we are relying on has never been more critical.

The learning health system (LHS)^4^ community has taken up the challenge of bringing the complex relationship between clinical research and practice into this brave new world. At the heart of the LHS vision is the notion of routine capture, transformation, and dissemination of data and knowledge, with various use cases, such as clinical studies, quality improvement initiatives, and decision support, constructed on top of specific routes that the data are taking through the LHS. These processes need to be mirrored by routine availability of trust information at each step of the process, embedding auditability and transparency in the very heart of the LHS.

This challenge is very timely, with the scientific community steadily becoming more aware of the fundamental problems in the way research is reported and results submitted to scrutiny in the postpublication stage.^5^ Reasons for this are complex and interleaved, including positive bias, intractable analyses, and pressure on journals and authors to constantly deliver groundbreaking research. Still, a consensus is arising that data‐driven solutions are the way to ensure correctness of science, making the LHS community, with its data focus, ideally positioned to spearhead this drive for improvement in medical research.

This paper shall review the main reproducibility challenges that affect medical research, before discussing the concept of data provenance as a way of embedding reproducibility into the LHS. The experiences of the TRANSFoRm project will be presented as an exemplar on how to incorporate provenance into 3 LHS use cases: epidemiological research, randomized controlled trials (RCTs), and diagnostic decision support. Finally, the impact of such step change will be discussed and directions for future research presented.

## 2 REPRODUCIBILITY CHALLENGES

While reproducibility has always been at the core of scientific method, it was only with the digitalization of the research task that it has become possible for external scientists and teams to attempt to fully reproduce research findings in‐house, using identical software tools, and data, when available. Two landmark studies that established the scale of the reproducibility crisis came from pharmaceutical industry teams looking to validate details of published findings before dedicating resources to produce them. A study by a team from Bayer showed that only 25% of 67 examined academic papers could be replicated.^6^ Meanwhile, scientists from Amgen looked at 53 preclinical oncology studies published between 2001 and 2011 and found that only 6 (11%) could be robustly replicated, with the irreproducible studies found to be attracting more citations than the reproducible ones.^7^

The problem is by no means restricted to preclinical studies, even though their increased reliance on computational instruments makes them easier to spot and test. The investigation of Young and Karr^8^ looked into 12 randomized clinical trials testing 52 observational claims and failed to reproduce a single one. Open Science Collaboration^9^ described the replication of 100 experiments reported in papers published in 2008 in 3 high‐ranking psychology journals. Assessing whether the replication and the original experiment yielded the same result according to several criteria, they found that only about one‐third to one‐half of the original findings were also observed in the replication study. Most recently, a random sample of 441 journal articles from biomedical journals from between 2000 and 2014 was studied, and it was found that none made all their data available, only one provided a full protocol, and the majority did not disclose funding or conflicts of interest.^10^ The cost of irreproducible research in life science is estimated at $28 billion per year in the United States, with a quarter of that sum attributed to data analysis and reporting.^11^

Lack of reproducibility creates translational problems on two fronts. Findings in basic and preclinical research that are supposed to set the agenda for the clinical studies and drug development are often poor predictors for success in the clinic.^12^, ^13^, ^14^ At the other end of the research spectrum, drugs with promising results in clinical trials are sometimes found to be underperforming in real‐world conditions, a concept referred to as the *efficacy‐effectiveness gap*.^15^ While the lack of transparency and insight into trial design and execution is by no means the only contributing factor to this phenomenon, it is a significant contributor.^16^

A number of institutions have emerged that are dedicated to promoting reproducible practices in scientific research, such as the Center for Open Science in the United States, *
https://cos.io/
 which provides free and open services to increase inclusivity and transparency of research. Meta‐Research Innovation Center at Stanford †
http://metrics.stanford.edu/
is dedicated to building cross‐disciplinary collaborations with the view of improving research practices across biomedical disciplines. The United Kingdom's Software Sustainability Institute ‡
http://www.software.ac.uk
 is focusing on the role of software in research reproducibility and promoting best practices in documentation, version management, release procedures, licensing, and archiving.

### 2.1 Inadequacies of publishing culture

So as to adequately address the reproducibility failings, some elements of the publishing culture need revisiting to increase transparency and traceability. A joint statement by editors of *Science and Nature*, following a workshop organized by the National Institutes of Health, highlighted the issue.^17^, ^18^ With regard to postpublishing guidelines, several common pitfalls in the process have been noted, including reluctance in publishing retractions, imposing fees for retracting articles or publishing comments challenging the published articles, and not providing mechanisms for access to raw data.^5^ Proposals have been made that the research teams should curate their data and software so that it is readily available, and its scrutiny should form a required step of journal peer review, eg, in Center for Open Science's Transparency and Openness Promotion Guidelines^19^ that have been endorsed by 538 journals at the time of writing.

To further these aims, the editors of the leading medical journals in International Committee of Medical Journal Editors made a proposal in the early 2016 to make public sharing of data gathered in clinical studies as a condition of publishing the results in those journals. The data concerned comprises deidentified individual‐patient data underlying the results presented in the article, tables, figures, and appendices or supplementary material, including necessary metadata.^20^ This proposal has not been without its critics though, with access to data being a particular point of contention. A recent editorial in *New England Journal of Medicine* 21 raised concerns about unsupervised use of data by teams that lack understanding of the data and ethical and regulatory mechanisms around it, and the emergence of a new class of “research parasites” that focuses on finding errors in published research. The use of term created a significant backlash in the research community, which prompted a response by the journal.^22^

### 2.2 Reproducibility in the LHS

Relying on the routine processes that both depend on research findings and produce further data for research, LHS is particularly vulnerable to failings in the quality of its research. Being a systemic change across multiple settings, it requires demonstrable trust in its every segment. We define 4 levels of reproducibility within the context of the LHS:

1. *Auditability* allows the research to be scrutinized according to some predefined methodology. A certain subset of research information is made available for further investigation.
2. *Traceability* establishes an unbroken chain of transformations that data underwent from its capture to its contribution to research findings.
3. *Replicability* offers researchers the ability to repeat the experiments and findings with identical tooling on original data.
4. *Reproducibility* asserts that the scientific result can be independently confirmed on new data.

It would be wrong to assume that replicability is a necessary precondition for reproducibility. Indeed reproducibility requires that findings are robust enough to survive minute changes to experiment design, whereas replicability avoids them by definition.^23^, ^24^ However, it is the role of traceability to establish exactly what happened during the experiment and provide reassurance that sources of variability are indeed scientifically insignificant and that the abstracted principles (eg, *P* values and confidence intervals) are sound. Thus, it is traceability that is central to establishing transparency and trust in the LHS processes. It is worth noting that these issues do not apply exclusively to research and that the same principles are applicable to routine quality improvement initiatives, which form another important part of the LHS.^25^

## 3 DATA PROVENANCE

A necessary precondition for improving the reproducibility of medical research is to increase the transparency of the research process, by including minute details that will allow subsequent investigators to fully understand what was actually done. Reporting standards for cohort studies^26^, ^27^ or clinical trials^28^, ^29^, ^30^ have long been the traditional means of providing this level of rigor in medical research, but they are inadequate in a data‐driven LHS, since they can only provide auditability, often at significant resource cost, but cannot establish transparency, much less traceability, replicability, or reproducibility. Manually producing standardized reports from a combination of notes and a collection of software artifacts introduces space for mistakes and omissions, exacerbated by the natural tendency of researchers toward observational and cognitive bias.^31^ Furthermore, such reports are typically not included with the original research publication and thus not readily available to readers.

Simply put, provenance describes what happened. W3C defines provenance as a form of contextual metadata “that describes entities and processes involved in producing and delivering or otherwise influencing that resource. Provenance provides a critical foundation for assessing authenticity, enabling trust, and allowing reproducibility.”^32^ The Office of the National Coordinator for Health IT describes it as “attributes about the origin of health information at the time it is first created and tracks the uses and permutations of the health information over its lifecycle.”^33^

Data provenance technologies can provide traceability to the LHS by automatically capturing the trace of the research task and resulting data in a uniform and domain‐independent way, thereby facilitating reproducible research. The concept originated in the eScience and cyber‐infrastructure communities, as means of capturing the exact parameterizations and configurations of scientific workflows that produced a particular data set.^34^, ^35^ As the number of implementations grew, the W3C developed the PROV interoperability standard^32^ that models provenance data as graphs where nodes represent data *entities*; *activities* produce and use those entities; and *agents* are actors that control these activities, with graph edges denoting the relationships between the concepts^36^: was controlled by, used, was generated by and others. While provenance data are not always stored in graph databases, this model conceptually implies that the provenance questions of interest require both traditional item‐based querying and exploratory analysis, whereby the researcher can browse the relationships between entities to find the answer.^37^ An example of a provenance graph can be seen in Figure 4, with entities denoted in blue, activities in red, and agents in yellow. The graphs are read in the direction of the arrows; thus, in the figure, the *Query Result* entity on the right was generated by the *Execute Query* process that used the *Data Collection Query* entity, which has its own further history recorded.

While provenance has a significant role to play in achieving reproducibility in the LHS, there are still gaps in its implementation methodology that stem from provenance having emerged from computational fields with standardized software architectures. Provenance adoption in noncomputational disciplines, such as the LHS, presents several challenges:

1. Problem domains, such as medicine, have *established software ecosystems* that cannot be easily replaced with provenance‐enabled tooling without major investment and disruption.
2. Motivating such change can be difficult, particularly if the immediate benefit is unclear, and without example provenance data for a particular problem domain, it is *difficult to demonstrate the capabilities of provenance analytics*.
3. Existing provenance models provide a common syntax for representing provenance, but creating semantically rich provenance models requires *involvement of domain experts*.

Furthermore, the specific goals of LHS being adaptable, self‐improving, stable, certifiable, and responsive^38^ introduce additional desirable features in a provenance solution.

1. *System transparency.* The black‐box approach and lack of transparency result in the lack of trust and are cited as two of the main reasons behind the poor take‐up of medical software and decision support systems (DSSs) in particular.^39^ Therefore, in a provenance‐enabled architecture, activities related to usage and generation of data need to be readily available for users to review.
2. *Auditability of actions.* The system must enable the user to look up an action performed in the system and find all the relevant detail about how it was made—data sets used, exact versions of software tools, and human actors involved. The level of detail captured must be validated against the required audit standard.
3. *Understandability of data.* The provenance metadata that is captured about the workings of the system not only needs to be accessible to the users (clinicians, auditors, researchers, patients) but also has to rely on standardized concepts expressed in terminologies that the users are familiar with.
4. *Validation readiness.* So as to guarantee that the provenance metadata being captured is at the right level of granularity and encompasses all the necessary features, the structure of the provenance data needs to be modeled and verified separately from the software implementation.
5. *Privacy and security.* Traditionally, security logs have been used to keep track of what is going on in the system and investigate any inappropriate actions. The provenance model needs to go beyond that and be able to demonstrate that a data set is never used contrary to its ethics and privacy constraints.
6. *Scalability.* The system must be able to scale up in line with the expected usage volume, so the provenance store needs to be appropriately specified to cope with accumulation of usage data over time.

We shall now describe how the provenance infrastructure in 1 large‐scale LHS project, TRANSFoRm, addressed these issues.

## 4 IMPLEMENTATION OF PROVENANCE INFRASTRUCTURE IN TRANSFORM

The TRANSFoRm project §
www.transformproject.eu
was funded under EU Framework Programme 7 to develop a common digital infrastructure for LHS applications, with the aim of integrating the data and workflows of clinical and research domains in primary care. The project outputs include methods, models, services, validated architectures, and clinical demonstrations of software to support this integration. The architecture that was developed is generic^40^ and was applied across the project's 3 demonstrators: genotypic‐phenotypic observational studies, RCTs, and diagnostic decision support.

**Figure 1:**
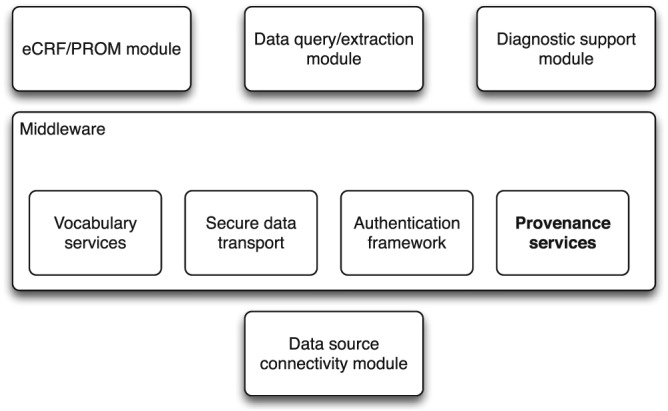
Overview of TRANSFoRm software components

The TRANSFoRm software ecosystem, shown in Figure 1, comprises front‐end tools that rely on a set of generic middleware components, secure data transport, authentication, semantic mediation, and data provenance, which provide essential shared functions for the LHS applications built in TRANSFoRm.

**Figure 2:**
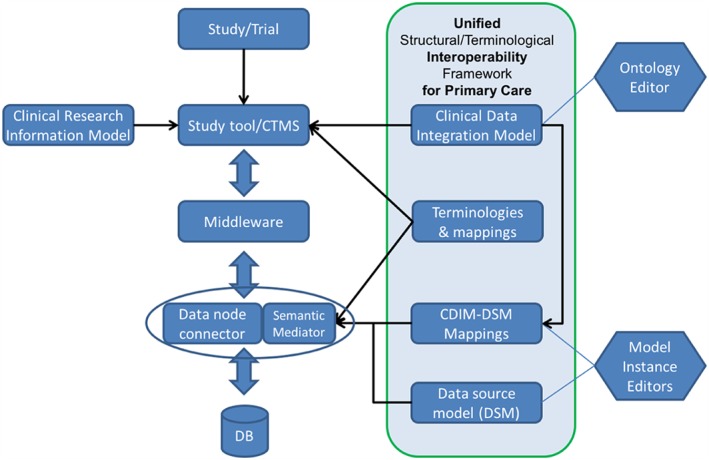
TRANSFoRm semantic stack. Study concepts are expressed in the clinical research information model, with data elements defined in the clinical data integration model (CDIM) and mapped onto the local database structures defined in the data source model (DSM), using local data node connector and semantic mediator components. CTMS indicates clinical trial management system

One of the goals for TRANSFoRm was to provide maximum flexibility, presenting the lowest possible barriers to entry for integrating electronic health record (EHR) systems and data sets, reusing whenever possible the existing data standards and methods for managing heterogeneity between data sources. The data used in the LHS reside in multiple repositories, differing in structure and terminology, bringing the need for a generic mechanism for mapping TRANSFoRm queries onto individual data sources. This was delivered using a semantic mediation approach,^41^ combined with a standard data connectivity module, as shown in Figure 2, which illustrates the translation process for observational studies and RCTs. Clinical concept data elements were modeled using the clinical data integration model (CDIM) ontology,^42^ and local data source models with the LexEVS tool used to support binding of terminology terms to CDIM expressions. The research processes were modeled by the clinical research information model (CRIM), which, in conjunction with CDIM, enabled a 2‐level archetype to be defined for each required data element in the 3 use cases.

On top of these shared components, 3 application specific tools were built to support the use cases: epidemiological study query workbench, clinical trial data collection and monitoring tool, and a diagnostic support plug‐in for EHR systems. The *query workbench* allows researchers to design their queries from multiple data sources and translate their clinical terms into a list of corresponding concepts from standard terminologies and classifications supported by the systems they are working with. The queries are dispatched to the data sources via the middleware to the local data node connector that sits at the data source and translates the generic CDIM‐based query into a local representation using the semantic mediator component and subsequently presents that locally interpretable query (patient counts, flagging patients, or data extraction) either to the data source directly or to a human agent for final approval, before returning the result. The *RCT module* comprises computerized trial definitions using extended CDISC's SDM/ODM standards, with automatically generated electronic case report forms (eCRFs) and Patient Reported Outcome Measures (PROMs). The former are filled in via a web browser by the clinician, while the latter are completed by the patients using either web or mobile devices. The tool integrates with the EHR to perform patient eligibility checks and enrolment, and prepopulation of eCRF data and to store a copy of study data in the EHR. The TRANSFoRm Study System coordinates study events and data collections across multiple clinical sites. Finally, the *diagnostic decision support tool* is embedded into the EHR and suggests to the clinician the diagnoses to consider, based on the patient record and presentation cues entered. Recommendations are generated by the central evidence service using rules stored in the clinical evidence repository and annotated with levels of support and confidence for the presenting case. The coded evidence cues and current working diagnosis can be saved back to the patient EHR.

**Figure 3:**
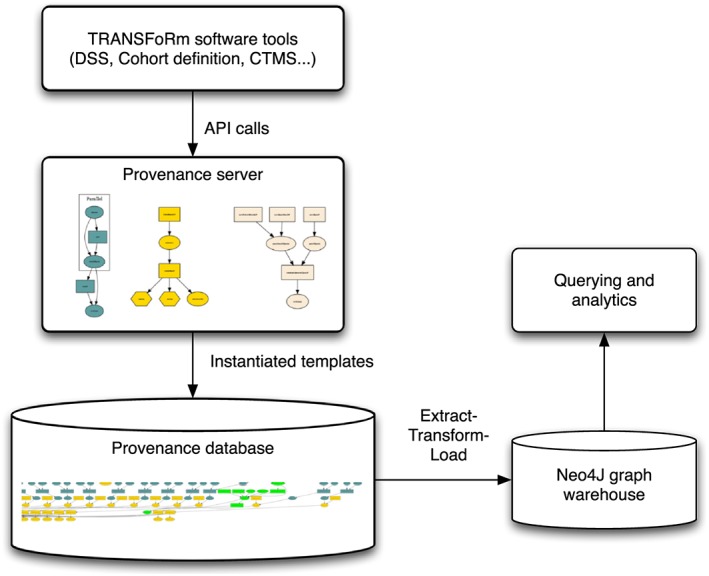
Provenance architecture in TRANSFoRm. Software tools are agnostic to the underlying provenance representation and invoke API calls that match some provenance template in the provenance server. Template is then instantiated into a provenance graph fragment with appropriate ontological annotations and persisted inside the relational database. The database is ETL‐ed into a Neo4J graph data warehouse, which is used for querying and analysis. API indicates application programming interface; CTMS, clinical trial management system; DSS, decision support system

Data provenance capture in TRANSFoRm implements traceability across these 3 use cases, which is necessary both to support trust and transparency and to enable learning and improvement in LHS processes. The need to capture provenance data from a number of heterogeneous data sources, without relying on each software tool to write its own provenance code, was addressed by the concept of *provenance templates*. Introduced by Curcin et al,^43^, ^44^ these are abstract constructs, which can be instantiated into concrete provenance graph fragments, that are added to the existing provenance graphs. The fragments are defined in terms of meaningful operations within the LHS problem domain, expressed using concepts taken from a relevant domain ontology (eg, CRIM) and that are mapped onto the provenance ontology PROV‐O.^45^ The choice of domain ontologies and their mapping onto provenance concepts also determines the granularity of captured provenance. Having established a set of meaningful actions that software tools in the domain perform, reflected in the templates, a service interface was defined for those tools to invoke and capture provenance without needing to undergo major redesign, as shown in Figure 3. While all the nodes and edges in provenance graphs are annotated with ontological concepts, for clarity, the images in the following sections use human‐readable labels derived from the concepts and identifiers.

The starting point for defining the provenance use cases was to express the use case requirements as a series of provenance‐related questions. We list these generic provenance questions for each use case to describe the provenance information that we require to be automatically recorded and available about the LHS and then show how it was implemented in TRANSFoRm.

### 4.1 Use case: epidemiological studies

The provenance challenge in the observational domain is to ensure that the queries used to extract data were aligned with the study protocol, and that any customization required by the data sources was correct and is available for auditing. Further useful feature is to track the popularity of individual data sources and how they are being used. Thus, within the context of cohort studies from distributed data sources, provenance information should allow us to find out the following:

- What was the exact query used in each database to select cases and controls for a study?
- How was a particular data extraction query modified before its final form, and what were the performances of discarded versions?
- Which data items came from which data source?
- Which are the most useful data sources across multiple studies?

This information is typically not readily available, particularly in a distributed query scenario, with the researchers having to rely on local logs that need to be interpreted and combined to provide full information, which adds to the resource cost of the study, potentially presenting a major problem given the relatively tight financial constraints on observational studies compared with clinical trials.

The TRANSFoRm epidemiological use case was implemented using a front‐end Query Workbench that researchers used to define their queries and send them through the middleware infrastructure to data sources. Three types of queries are supported (counts, flagging, and data extraction), each of which is expressed in a generic query model using generic CDIM representations of medical concepts inside the queries, such as inclusion and exclusion criteria and data fields to be retrieved. Once the query arrives at the data provider's site, it gets translated into the local representation, verified and authorized by the local data controller, and executed against the database.

The provenance data that were captured in the use case, based on a set of templates, covered the following: users logging in and being authenticated in the system, creation and editing of queries, and execution of queries against the databases. All 3 of these scenarios were performed by different tools, first by the authentication system, second by the Query Workbench, and the third by the Data Connector, with the TRANSFoRm template‐based provenance service collecting the data.

**Figure 4:**
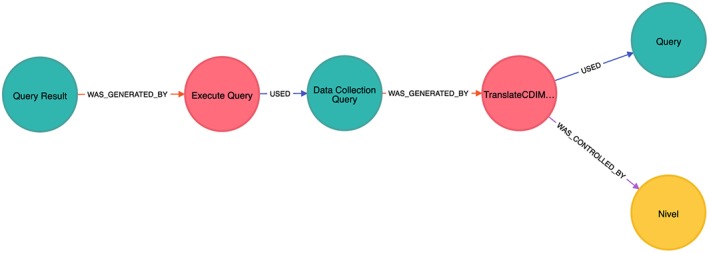
Provenance graph depicting an execution of a data extraction query in the epidemiological use case. Blue nodes denote entities, red nodes are processes, and yellow nodes are agents. The graph is read from the bottom node, which is a query result generated by the query execution process, which used a data collection query that was generated by the translation process, controlled by the local data provider (Nivel) and that was obtained from a generic query

The provenance graph fragment shown in Figure 4 contains the trace of the third scenario, visualized in the Neo4J database, with blue nodes as entities, red nodes as processes, and yellow nodes as agents. The Query Result entity at the bottom of the graph was produced by the Query Execution process, which used the Data Collection Query containing the actual SQL query, which was in turn obtained from the Translation process using a generic CDIM query and controlled by the data controller at the data provider's site (Nivel ¶
www.nivel.nl
in the example shown). The node labels have been generated from the ontological categories and values that all nodes have been annotated with, taken from TRANSFoRm's CRIM. The freedom to annotate the nodes with categories and values as per the requirements of the audit trace needed is a powerful mechanism for producing traces targeted for particular types of reports.

### 4.2 Use case: clinical trials

Food and Drug Administration's final guidance on Electronic Source Data in Clinical Investigations^46^ encourages use of electronic source (eSource) data in the conduct of clinical trials intended for inclusion in investigational and new drug applications, with the view that Electronic Data Capture from both devices and trial participants has the potential to improve the reliability, quality, traceability, provenance, and integrity of data from electronic source to regulatory submission. However, this effort is hampered by the lack of framework for capturing this metadata across various eSources.

In addition to providing an auditable research trail, the aim of using provenance in clinical research is to achieve deeper understanding of the trial characteristics and how it impacted its performance. So some of the items that can be addressed using RCT provenance data are as follows:

- Which trials had the largest/smallest number of consent rejections?
- Which trials failed to recruit patients for whatever reason?
- Which eligibility criteria were too restrictive in terms of the numbers of recruited vs expected patients?
- Which EHR‐s/locales were best at recruiting patients?
- What eCRF items in various sites were consistently not extracted from the EHR but had to be entered manually?
- For a particular study participant, list all versions of a specified eCRF, together with the date and the people involved in each modification.
- Retrieve details of informed consent for a patient who filled in a specific eCRF.

While some of these questions are answerable using the standard reporting functions of clinical trial management system tools, having a single audit repository becomes advantageous once several eSources and associated software tools start participating in the trial process, eg, clinical trial management system, EHR, mobile data collection tools, and EHR adapters, and all their entries can be queried jointly.

In its Randomized Controlled Trials use case, TRANSFoRm implemented an EHR‐driven clinical trial system, in which the EHR system was used to significantly reduce the effort in running clinical studies by automatically checking patients for study eligibility, using EHR data to partly fill eCRFs, and coordinating the study workflow including mobile data collection of PROMs. The resulting system was used to run studies with over 600 patients in 4 European countries^47^ and was validated for Good Clinical Practice.^29^ Traceability of such system is of essence so as to understand, evaluate, and potentially improve the trial design. This requires studying minute details, eg, eligibility criteria encodings, how they were applied to individual patients who presented to the clinician, data extracted from the EHR systems, data collected through eCRFs, and the analysis performed on the collected data.

Similarly to other use cases, assembling the trace of the entire process used provenance data captured from multiple tools: the TRANSFoRm Study System, EHR system, and the eCRF/PROM data collection tools. The provenance traces for RCT were significantly more complicated than those for the other 2 use cases, however, since they had to cover patient eligibility checks, consenting, randomizations, and data collections through eCRFs and PROMs, together with the interactions between eCRFs and the host EHR.

**Figure 5:**
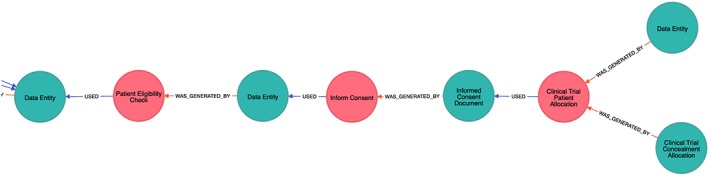
Fragment of a provenance graph depicting one patient's data record being checked for eligibility, patient being consented and randomized, and having the first study form created. Blue nodes denote entities and red nodes are processes

A fragment of the collected RCT provenance data is shown in Figure 5. As in the previous example, red nodes denote activities and blue are entities. The graph shows one patient's data record being checked for eligibility, patient being consented and randomized, and having the first study form created. The implementation uses the provenance ontology for randomized controlled trials to provide trial‐relevant semantic annotation on the provenance nodes,^43^ and nodes are labeled with concepts from provenance ontology for randomized controlled trials. The values of the nodes contain various information relevant to the concept, eg, eCRF document definition in ODM, EHR version, and timestamp. It is important to note that no patient identifiable information was stored in the provenance logs as these were kept separately from the research database, so the eCRF provenance entity contains the eCRF identifier in the TRANSFoRm Study System, but not the actual data stored inside.

### 4.3 Use case: decision support

The notion of trust is central to the LHS vision of routine capture, transformation, and dissemination of data and resulting knowledge and an essential aspect of that trust is to ensure transparency at each step of the process. When applied to DSSs, this translates to the ability to readily demonstrate the clinical reasoning that was performed in a clinical encounter, together with the recommendation received and a full trace from that recommendation back to the rules applied to produce it. Similarly to the RCT case above, although EHRs typically have auditability features, third‐party plug‐ins and DSS‐s often do not, questioning their ability to meet medical device certification standards.

The key provenance questions that are relevant to DSSs are as follows:

- Which clinician used the decision support tool to make a specific diagnostic recommendation for a specific patient at a specific point in time?
- What clinical evidence cues supporting diagnosis of a particular diagnostic condition were matched to a particular patient evidence set as part of an evidence comparison process that was run at a particular point in time?
- Which rules are most frequently fired for a particular set of symptoms?
- Which rules are never getting used?
- In which decisions was a particular rule/guideline applied?

Generic DSSs use a standardized model representation to encode the rules and guidelines that they implement. Should one such rule be found to be invalid or potentially harmful, it is important to be able to trace all usages of that rule, which is sometimes referred to as *taint analysis*. As an added benefit, accumulated provenance traces describing rule usage form a potentially valuable resource when assessing rule performance in practice.

In its third use case, TRANSFoRm has developed a prototype next generation diagnostic DSS. The tool is driven by clinical knowledge obtained through a web service–based clinical evidence repository and is embedded into a family practice EHR system (InPractice Systems Vision 3 EHR). The user enters observed patient cues with potential differential diagnoses being dynamically ranked, the cues are sent to the recommendation engine, and suggested diagnoses are returned. Upon exiting the tool, a working diagnosis can be confirmed, and the coded evidence cues and current working diagnosis can be saved back and recorded for future reference in the patient EHR.

Two DSS scenarios were identified as being relevant to capture through provenance metadata. The first use case describes the necessary provenance collection requirements for evidence production, generation, and update of evidence either through manual evidence update or through evidence generated automatically from the use of data mining tools. The second use case supports provenance collection during evidence consumption and subsequent clinical recommendation provided by the deployed evidence repository accessed by the decision support tool itself.

**Figure 6:**
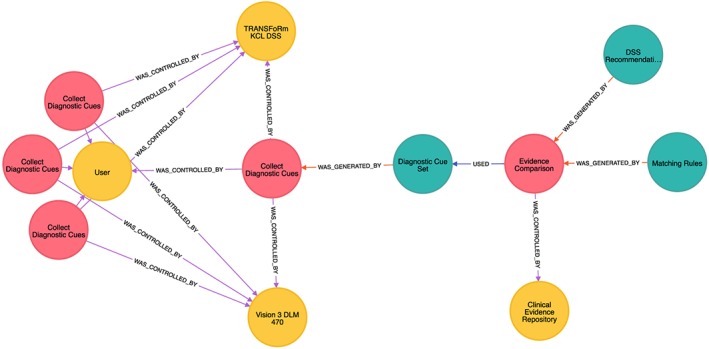
DSS provenance graph depicting the origin of a recommendation made by the system (top right). Its origins are traced back via the evidence comparison performed by the rule base and the patient cues presented all the way to individual EHR (InPS Vision) and DSS software instances used. DSS indicates decision support system

An example provenance trace from the second DSS scenario is shown in Figure 6, with blue nodes representing entities, red nodes activities, and yellow nodes agents, as described previously. This provenance trace shows the history of the DSS recommendation entity on the far right, detailing how it was produced by a clinical evidence comparison process, which compared cues obtained from the patient with the evidence residing in the clinical evidence repository (yellow actor node), with the full details of the cue collection and the DSS and EHR systems captured as well. The ontological concepts annotated onto the nodes and used to derive labels are taken from the TRANSFoRm's clinical evidence model ontology, containing relevant DSS concepts, and aligned with the constructs in the PROV model, in the same manner as in the other use cases.

## 5 DISCUSSION

To implement traceability, TRANSFoRm developed a generic solution for capturing provenance data in LHS applications, based on provenance templates and use of domain ontologies to attach precisely defined meaning to the collected metadata and thus addressed the 3 provenance challenges identified earlier. The use of a RESTful service application programming interface that hid the provenance‐specific detail behind high‐level service calls minimized the effort needed to connect software components to the provenance system. Using example provenance graphs, based on the abstract template constructs, enabled the creation of prototype reports and analytics to discuss with the end users. Such early prototypes can serve as powerful incentives for provenance adoption. Finally, the use of concepts taken from the relevant domain ontology when defining templates ensured that the structure and level of granularity of provenance metadata have been understood and agreed by the domain users. With regard to the further features mentioned in Section 3, our solution offers full transparency and auditability of data entities and their histories, allowing for demonstrable adherence to security policies, while the understandability of captured data is provided through use of domain ontologies, which in combination with the templates facilitates the validation task. The provenance work in TRANSFoRm was a pioneering effort in the field, and the next step is to conduct a detailed evaluation in each of the 3 problem domains by formally validating the template instruments and collected data against relevant standards and investigating the scalability aspects, with projects already underway in the decision support and clinical trials domains.

Provenance solutions can of course be implemented locally, so that they are specific to problem in hand. However, a generic approach, such as the one used in TRANSFoRm, enables us to reuse the same infrastructure for multiple applications so that, for example, a system used for clinical trials could also support the observational studies performed in the same institution. Apart from financial benefits, this approach supports a greater degree of connectedness between studies, facilitates long‐term data reuse within an environment, and increases the quality of metadata available about the institutional data sets, all of which become further incentives for provenance adoption.

Closely related to the issue of trust that has been discussed is the concept of security of provenance data. Ideally, we would want the provenance traces to act as a central piece of metadata about the LHS task observed, amenable to reporting the task to a variety of stakeholders, providing both detailed and high‐leveled views depending on the audience. For tasks involving sensitive data or steps, mechanisms have been developed to abstract portions of provenance graphs that should not be made accessible to certain users.^48^, ^49^ Thus, if we were looking into provenance of a clinical trial process, the researcher may be able to see the full detail of the patient recruitment and eligibility checks performed on each patient, while the provenance version published with the paper would only contain the study eligibility criteria used and recruitment outcome, without revealing individual patient's profiles.

### 5.1 Related work

The full provenance architecture and the details of the template model used in TRANSFoRm are currently submitted for publication and are under review. The templates that the solution is based on are similar to the efforts of the team at University of Southampton,^50^ with the main difference being that their work is better suited to atomic instantiations, where each template is immediately instantiated in full, while the TRANSFoRm model allows for variable repetitions (eg, sequence of edits to a study protocol). The PRIME methodology^51^ covers the life cycle of provenance model design, from use case specification to identification of actors, processes, and information flows, but it stops short of defining the architecture for provenance capture, the joint work on which is underway. Related to our use of ontologies for constraining provenance artifacts is the wider effort in the use of ontologies as part of the software engineering process,^52^ eg, through translations between ontologies and UML constructs.^53^ A broader overview of provenance implementation issues in biomedical research can be found in the work of Curcin et al.^44^

Recently, the DPROV initiative ‖
http://wiki.siframework.org/Data+Provenance+Initiative
has been working on aligning data provenance with the HL7 and FHIR protocols, with the goal of identifying opportunities within CDA R2 where basic provenance information about clinical (and other care related information) can be integrated, eg, who created it, when was it created, where was it created, how it was created, why it was created, and what action was taken to produce the information captured, thus enabling detailed audit of the data entry process.

Deciding the level of granularity of provenance capture is a recognized problem in the field. Indeed, there are infrastructures that collect finely grained provenance, on the level of the operating system (Hi‐Fi,^54^ SPADE,^55^ PASS,^56^ and PLUS^57^) or of individual programmatic scripts (noWorkflow^58^). In both cases, the scale of captured data and lack of semantics make the resulting provenance trails difficult to link to underlying research domain. Our approach minimizes the disruption required to instrument existing code by interleaving provenance‐specific elements into the code, in line with the principles of aspect‐oriented programming.^59^ An alternative approach is to reconstruct provenance from separately maintained logs,^60^ but this comes at the cost to the level of confidence in the resulting provenance data.

As part of the W3C PROV initiative, a comprehensive survey of available provenance implementations was assembled in 2013, which lists a wide range of provenance‐related software tools at various levels of maturity.^61^

### 5.2 Use of provenance for validation against standards

An important goal for the LHS community is to use provenance to demonstrate compliance of the software tasks executed with applicable standards and regulations. The most obvious example in the clinical trial domain is Title 21 of the Code of Federal Regulations; Electronic Records; Electronic Signatures (21 CFR Part 11)^28^ and Good Clinical Practice^29^ standards in the US and EudraLex Vol. 4 Annex 11: Computerised Systems in EU.^62^ Provenance can act as enabling technology to help software tools address the Technical Controls of 21 CFR Part 11 that regulate electronic records, namely,

- Discerning invalid or altered records.
- Generating accurate and complete records.
- Controlling task sequencing when event order is important (ie, operational checks).
- Protecting records throughout the record retention period.
- Generating an audit trail through the record retention period contains date/time of operator entries and description of actions taken and is cumulative.
- Limiting access to the system to authorized individuals
- Limiting access to system functions to authorized users (ie, authority checks).
- Limiting data input to authorized sources (ie, device checks).
- Protecting transmission of data from point of creation to receipt.

Addressing these in turn, (a) can be achieved by tracking access to each record and the user/actor who performed it. Analysis of captured provenance data can ensure that (b) is satisfied, although that can be checked on the main record repository as well. The sequence of steps executed, required in (c), can be proven using timestamped provenance traces and causality relationship between the nodes. (d) needs to be addressed at the data management level, where mechanisms such as nonrepudiation can be implemented, but provenance security techniques can be deployed to implement secure views on a single provenance metadata repository. (e) is ideally suited to being answerable using provenance audit trail since data provenance techniques allow all required information from multiple software and human actors to be placed in a single audit database using a uniform data model. (f) and (g) can be asserted by checking for the human agents that executed relevant processes in the provenance trace. Similarly, for (h), such checks can be applied to software agents. Finally, for (i), provenance provides an unbroken chain of actions and transformations that apply to the piece of data.

By extracting relevant parts of the provenance trace, reporting can be automated in accordance with the relevant standard, such as consolidated standards of reporting trials (CONSORT).^30^ Similar standards exist for reporting of cohort studies, such as Strengthening the reporting of observational studies in epidemiology (STROBE)^26^ and REporting of studies Conducted using Observational Routinely‐collected Data (RECORD),^27^ and the same principles can be applied to them as well, as long as suitable ontological annotations are provided on the provenance traces.

A significant advantage of data provenance technologies over standard logs for validation purposes is its ability to provide uniform history record across multiple software tools, thus creating a single audit trail to be examined, with consistent timestamping and simplified security and hosting policies. When used for reporting and audit purposes, it is important to validate the provenance captured against the structure, content, and granularity required by the applicable standards. Mechanisms for specifying the structure of provenance traces, like provenance templates used in TRANSFoRm, allow conformance to standards to be established at design time, facilitating validation. In such scenarios, it is also useful to treat provenance data stores as an adjunct part of the study database to ensure that relevant study information can be either replicated or referenced from the provenance entries.

## 6 CONCLUSION

Looking back at the 4 levels of reproducibility in the LHS introduced earlier, we have established that data provenance provides us with the traceability of data and processes. If those provenance traces contain sufficient detail and are using the correct domain conceptualization (ie, through well selected ontologies), they can be used to guarantee auditability as well. Replicability is more easily achieved in computational use cases with few nondeterministic elements, such as a diagnostic recommendation, or a data extraction and subsequent statistical analysis. In such scenarios, provenance, together with original data and software used, guarantees replicability. Finally, reproducibility is facilitated by the presence of full provenance information, ensuring detailed understanding of what occurred in the observed task, and also the methodological soundness of the techniques applied.

Ultimately, LHS aims to scale up health systems, and consequently the associated research that health systems are built upon. If this scaling up is to succeed, we have to embed mechanisms to verify trust in the system inside our research instruments. In the research world increasingly reliant on electronic tools, provenance gives us a lingua franca to achieve traceability, which we have shown to be essential to building these mechanisms. To realize the vision of making computable provenance a feasible approach to implementing reproducibility in the LHS, we have to provide viable mechanisms for adoption. These include defining meaningful provenance models for problem domains and also introducing provenance support to existing tools in a minimally invasive manner.

The applicability of data provenance to the challenges facing the LHS was demonstrated in a provenance infrastructure that was implemented in the TRANSFoRm project in 3 distinct LHS domains, those of observational studies, clinical trials, and DSSs. The challenge now is to address the provenance gap that exists between the provenance metadata collected and the reporting requirements of different domains and perform a full evaluation in each domain, which will require a joint effort by a range of stakeholders, including medical scientists, informaticians, publishers, and regulators. However complex and challenging, this work is essential if the quality of translation from research into practice in the LHS is to improve with the growing volume of data and research, rather than deteriorate and get lost in the noise.
